# Effectiveness of whole grain to body weight and cardiometabolic risk in adults with obesity: a parallel randomised controlled trial

**DOI:** 10.3389/fnut.2026.1774209

**Published:** 2026-03-11

**Authors:** Yongjun Wang, Tingting Liu, Jiali Cheng, Jing Feng, Jie Ding, Ye Ji, Rui Zhao, Junsheng Huo, Zhaolong Gong, Qin Zhuo

**Affiliations:** 1 Key Laboratory of Public Nutrition and Health of National Health Commission, National Institute for Nutrition and Health, Chinese Center for Disease Control and Prevention, Beijing, China; 2 Department of Clinical Nutrition, Shandong Provincial Qianfoshan Hospital, The First Affiliated Hospital of Shandong First Medical University, Shandong, China

**Keywords:** body weight, cardiometabolic risk, foods, obesity, whole grain

## Abstract

**Background/objectives:**

Obesity is a global health problem associated with an increased risk of cardiovascular disease. Whole grains are thought to contribute to weight management and reduce cardiovascular risk factors.

**Methods:**

A parallel randomised controlled trial was conducted with 115 obese adults who were randomly assigned to one of two whole grain intervention groups (50 g and 100 g per day) or to control group.

**Results:**

The mean weight change from baseline to 12 weeks between the 50 g/d whole grain group and the control group was −2.0 kg (95% CI, −3.1 to −0.8), and −1.7 kg (95% CI, −2.7 to −0.6) between the 100 g/d whole grain group and the control group. Body-mass index (BMI), body fat and abdominal visceral fat also showed significant reductions in the whole grain groups compared to the control group. Fasting blood glucose and insulin resistance improved in both whole grain intervention groups. Additionally, triglycerides and total cholesterol levels and triglyceride glucose (TyG) index decreased significantly in the 50 and 100 g/d whole grain groups. Serum uric acid and homocysteine levels were observed in the 50 and 100 g/d whole grain groups with notable decreases compared to the control group.

**Conclusion:**

In adults with obesity, the whole grain intervention significantly improved weight loss and metabolic health. These findings support policies to increase whole grain consumption in the management of obesity.

**Clinical trial registration:**

www.chictr.org.cn, identifier: ChiCTR2300072952.

## 1 Introduction

Obesity is a multifaceted, progressive, and chronic condition that has been demonstrated to induce cardiovascular complications and elevate the risk of developing various pathologies, including type 2 diabetes, hypertension, certain forms of cancer, and even all-cause mortality (1). By 2035, it is estimated that approximately 3.3 billion adults worldwide will be overweight or obese, making it a major global public health challenge (2). Obesity has been shown to be associated with a multitude of factors, including genetics, environment, socioeconomic status, and lifestyle (3–5). The benefits of weight loss include improved metabolic state, a diminished probability of developing type 2 diabetes, a reduced risk of cardiovascular and renal diseases, and a decelerated process of ageing (6–9). Numerous strategies have been developed for the purpose of combating obesity and reducing body mass. These include adjunctive treatments such as anti-obesity medications (10–12) and surgical interventions (13, 14), which have been increasingly used in recent years. Nonetheless, it is widely acknowledged that lifestyle and behavioral modifications remain crucial components and fundamental principles in the management of obesity (15).

The improvement of dietary structure and quality is an integral component of the establishment of a healthy lifestyle. Whole grains are a constituent of numerous healthy dietary patterns. The increment of whole grain intake has been demonstrated to engender a wide range of health benefits, including a reduction in the risk of diabetes, cardiovascular disease, colorectal cancer and other cancers and all-cause mortality (16, 17). Furthermore, it is advantageous for healthy ageing (18) and weight management (19).

The relationship between consuming whole grains and successful weight management, as well as cardiovascular health, has been a subject of considerable research interest (20). Previous studies have demonstrated that whole grains are rich in dietary fiber, which can increase satiety and reduce energy intake (21). In addition, unlike refined carbohydrates, whole grains are low glycemic index foods, which helps lower blood glucose levels and increase insulin sensitivity, a major factor in weight gain in obese people (22). Whole grains are abundant in dietary fiber and polyphenolic compounds, which may improve the chronic inflammatory condition of obese individuals by enhancing intestinal health and reducing oxidative stress, thus supporting to weight management (23, 24). According to existing literature, dietary patterns are a key determinant of gut microbiota profiles. Specifically, whole grains have been reported to beneficially regulate gut microbial composition and enhance short-chain fatty acid levels, which are proposed to help in weight management (25). Considering healthy food for weight management, whole grain foods represent a promising dietary strategy for weight management. However, high-quality evidence base for this claim remains limited due to lack of randomised controlled trials investigating the effect of whole grain intake on body weight and cardiometabolic risk in obese adults. While meta-analyses have confirmed the benefits of whole grains, high-quality randomised controlled trials directly comparing different dosages remain limited for obese adults—the group with the highest metabolic risk and most urgent need for intervention. The heterogeneity of the whole grain interventions and the different times at which they were implemented confounded the evaluation.

Therefore, we conducted a randomised controlled trial to examine the effect of whole grain on body weight in adults with obesity through a whole grain dietary intervention. We also assessed the impact of whole-grain intervention on cardiometabolic risk.

## 2 Materials and methods

### 2.1 Study design

This randomised, parallel, controlled trial was conducted in obese adults. The trial protocol for the trial was reviewed and approved by the ethics committee of the National Institute for Nutrition and Health, Chinese Center for Disease Control and Prevention ethics committee (2023-002), and all participants provided written informed consent. The trial protocol was registered in the Chinese Clinical Trial Registry (registration number: ChiCTR2300072952).

### 2.2 Participants

This study was conducted from August 2023 to January 2024 in Jinan, China. Recruitment of participants was achieved through the distribution of promotional leaflets and posters, in addition to online recruitment advertisements. Participants were eligible for inclusion in the study if they were aged between 18 and 65 years and had a BMI within the range of 28–40 kg/m2. Individuals who were pregnant or intending to become pregnant in the next 3 months, those with gastrointestinal disorders, and those suffering from the following diseases were excluded: tumors, heart, liver, and kidney dysfunction, severe infectious diseases, and autoimmune diseases. Furthermore, participants with a history of allergic symptoms to whole grains or a daily intake of whole grains exceeding 100 g, a weight change of more than 3% in the last 3 months, or medication that may affect weight were excluded from the study. All research-related assessments were conducted in a hospital setting, while the intervention was implemented in participants’ daily home environments.

### 2.3 Interventions

During the 12-week study period, subjects in the whole grain intervention group were provided with whole grains foods, including whole grain steamed buns or whole grain rice, which were produced by Tongfu Group Co., Ltd. (Shijiazhuang, China). Whole grain steamed buns were primarily composed of whole wheat flour and wheat flour, while whole grain rice was formulated from brown rice, red rice, oats, and red quinoa. The raw materials and nutritional content of whole grain foods can be found in the Supplementary Table S1. Based on the recommended range in the Chinese Dietary Guidelines and aligned with intake levels from patterns like the Mediterranean diet, we set the two intervention doses at 50 and 100 g/d. Participants in the 100 g/d whole grain group were required to consume 100 g of whole grains, provided in the form of buns, rice, or a combination thereof. The intake target for the 50 g/d whole grain group was 50 g of whole grains daily. Both groups were required to replace an equal amount of their staple food with the given whole-grain staple food. Participants in the whole grain intervention group were requested to attend the site every 2 weeks to receive whole grain food for the subsequent 2 weeks and to return the whole grain food consumption record form to enhance compliance. Participants in the control group maintained their habitual dietary intake without undergoing any intervention. All three groups of participants established online follow-up and support groups via WeChat (Tencent, China). To facilitate accurate data collection, the research team provided each participant with a food diary form and an electronic scale at the beginning of the trial, and asked them to record detailed daily food intake for two consecutive weekdays and one weekend day in weeks 0, 6 and 12.

### 2.4 Outcomes and measurements

The primary outcome of the study was the difference in weight change among the three groups of participants from baseline to 12 weeks. The secondary outcomes encompassed a range of metrics, including changes in BMI, waist circumference, body composition (e.g., body fat mass, body fat percent, body lean mass, and area of abdominal visceral fat), glucose and lipid metabolism-related indicators, blood pressure, and cardiometabolic risk factors such as serum uric acid and homocysteine, all measured from baseline to 12 weeks.

Basic demographic information, lifestyle information (including smoking and drinking habits), physical activity, medical history and quality of life were collected by trained field investigators through a paper questionnaire. The level of physical activity was collected using the International Physical Activity Questionnaire (IPAQ), and the quality of life was assessed using the 12-item Short-Form Health Survey Questionnaire (SF-12). Standard procedures were employed to measure body weight, height, waist circumference, body composition, blood pressure, and heart rate. These measurements were taken from participants in a fasting state at baseline, week 6, and week 12. Body composition was assessed using the multi-frequency bioelectrical impedance analysis device InBody770 (InBody Co., Ltd., Seoul, South Korea). The extracted parameters included total body fat mass, percent body fat, lean body mass (including skeletal muscle mass), and visceral fat area, following the manufacturer’s standard measurement protocol. Measurements were conducted in the morning under standardized conditions (after an overnight fast, empty bladder, light clothing).

Venous blood samples were collected from participants following a 10-h overnight fast. Serum and plasma were separated by centrifugation and analyzed at the central laboratory of the National Institute for Nutrition and Health, Chinese Center for Disease Control and Prevention. All assays were performed using standardized clinical laboratory methods with strict internal quality control procedures. Specific methodologies were as follows: Fasting plasma glucose, total cholesterol, triglycerides, high-density lipoprotein cholesterol (HDL-C), and low-density lipoprotein cholesterol (LDL-C) were measured using enzymatic assays on a Hitachi 7,600 automated clinical chemistry analyzer (Hitachi High-Technologies Corporation, Tokyo, Japan). Glycated serum protein (GSP), glycated hemoglobin (HbA1c), serum homocysteine, and uric acid levels were quantified using an enzyme-cyclic assay on a clinical chemistry automated analyzer (enzyme reagents supplied by Beijing Leadman Biochemical Technology Co., Ltd.).

### 2.5 Harms

The intervention measures employed in this study have the potential to induce mild physical and emotional discomfort, including symptoms such as fatigue and indigestion. During the trial, potential adverse reactions were continuously collected and the health status of the participants closely monitored. Should any of the participants exhibit signs of serious or deteriorating conditions, the researchers reserve the right to either take appropriate measures or to cease the intervention.

### 2.6 Sample size

The present study constitutes a randomised parallel controlled trial. Participants were randomly assigned to one of three groups: 50 g/d whole grain intervention group, 100 g/d whole grain intervention group, or control group. The primary outcome measure was the difference in weight change from baseline to 12 weeks of follow-up or intervention. The findings from the literature review and extant research provide a foundation for interpreting the results (26). For instance, the mean weight change in the control group after 12 weeks was 0.21 ± 1.09 kg, the mean weight change in the 50 g/d intervention group after 12 weeks was 1.54 ± 1.64 kg, and the mean weight change in the 100 g/d intervention group after 12 weeks was 2.08 ± 2.05 kg. Assuming a two-sided alpha level of 0.05 and a power of 90%, a total of three groups of participants were allocated in a 1:1:1 ratio. Utilizing PASS 15 software (NCSS LLC., Kaysville, U.T., USA), it was calculated that there would be 30 cases each in the 50 g/d intervention group, the 100 g/d intervention group and the control group. Considering the anticipated rates of dropout and refusal, which were set at 20%, it was determined that a minimum of 38 subjects was required for each of the intervention and control groups. Consequently, it was determined that this experiment would necessitate the participation of at least 114 individuals.

### 2.7 Randomisation and blinding

In this study, researchers employed block randomisation (block size 6), generating random allocation sequences created with Microsoft Excel software to randomly assign participants in a 1:1:1 ratio to a 12-week intervention consisting of either 50 or 100 g/d of whole grains, or to a control group. Each participant will undergo a baseline survey in sequence once they have provided written consent, and after completion, they will be randomised into groups. The investigators responsible for the baseline survey and the staff members responsible for randomisation were unaware of the specific group assignments. Due to the differences in the intervention itself and the variety of food, it was not possible to mask the participants.

### 2.8 Statistical analysis

All analyses were performed following the intention-to-treat principle. The primary hypothesis was that participants in either whole-grain intervention group would achieve a greater reduction in body weight at week 12 compared to the control group. Secondary hypotheses pertained to between-group differences in cardiometabolic risk markers.

Generalized Estimating Equations (GEE) were used to analyze changes in outcomes across baseline, week 6, and week 12. This method accounts for within-subject correlation of repeated measures and provides robust estimates under the missing-at-random assumption. An exchangeable correlation structure was specified based on quasi-likelihood under the independence model criterion (QIC). The model included group (control, 50 g/d, 100 g/d), time (baseline, week 6, week 12), the group-by-time interaction, and the following pre-specified covariates assessed at baseline: age, sex, height, education, marital status, income, smoking status, alcohol consumption, physical activity, and parental history of hypertension, diabetes, or obesity. Covariates were selected based on their established clinical relevance to cardiometabolic outcomes. For the primary outcome (body weight), the significance of the group-by-time interaction at week 12 was evaluated. If significant, pairwise comparisons (50 g/d vs. control; 100 g/d vs. control) were conducted using post-hoc Wald tests.

All statistical analyses were conducted using R (http://www.R-project.org, version 4.3.1) and IBM SPSS Statistics for Windows, version 26.0 (IBM Corp., Armonk, NY, USA). All statistical tests were two-sided, and *p* values < 0.05 were considered statistically significant.

## 3 Results

### 3.1 Baseline characteristics of participants

**Figure 1:**
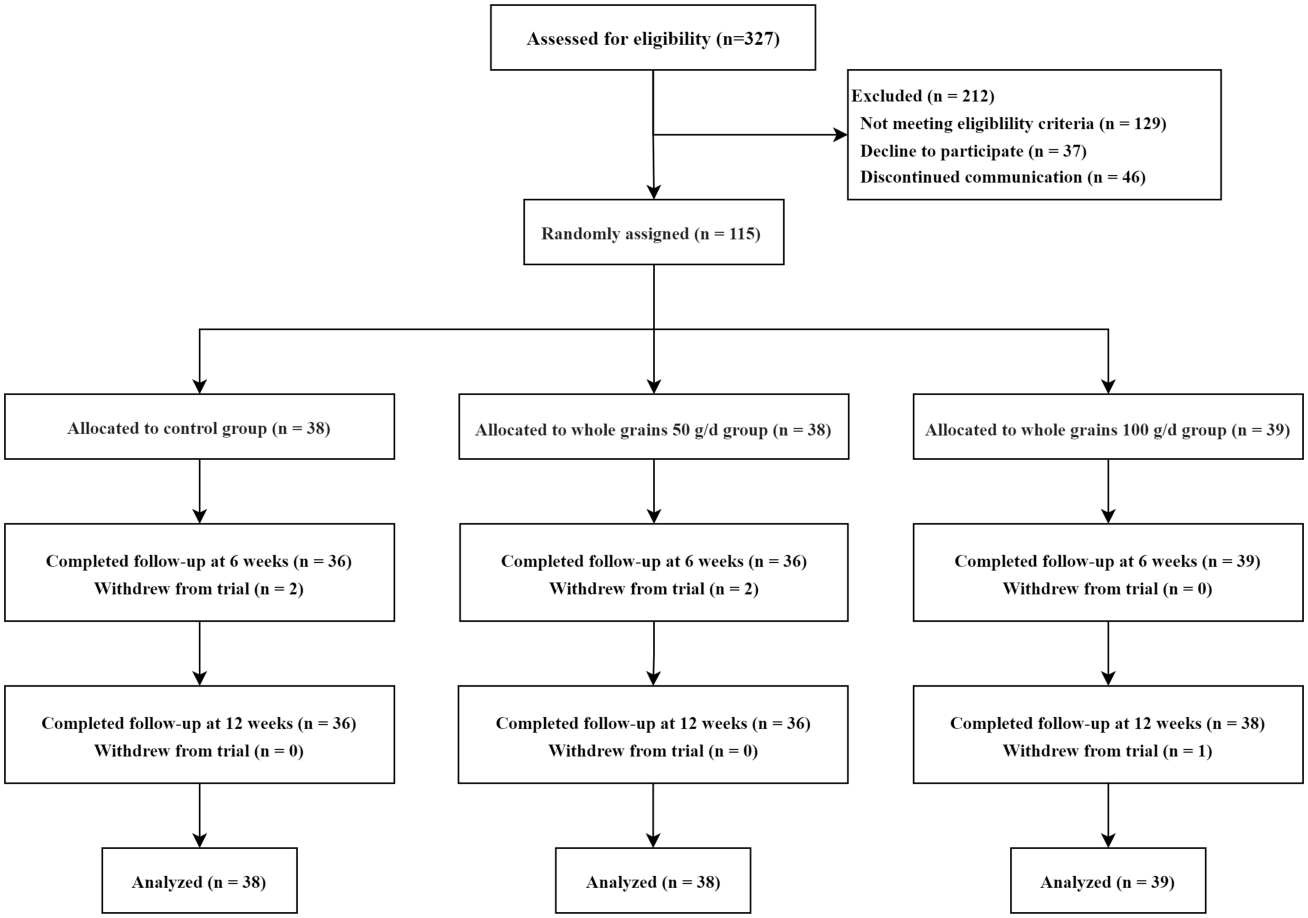
Study design and flow chart.

From August 2023 to October 2023, 327 adults expressed interest in participating in the trial. Of whose 129 were ineligible, 37 declined to participate, 46 could not be reached for further contact, and ultimately 115 adults were randomly assigned to three groups. The 50 g/d whole grain group comprised 38 participants, the 100 g/d whole grain group comprised 39 participants, and the control group comprised 38 participants. Of the 115 participants, 111 (97%) completed the 6-week follow-up, and 110 (96%) completed the 12-week follow-up. The detailed flow of participants through the trial is shown in Figure 1. Baseline demographic information is displayed in Table 1 and Supplementary Table S2. At the time of the baseline assessment, the characteristics of the participants in the three groups were similar. The average age of participants was 36 years (SD, 9), with 45% being female. The mean weight of participants was recorded as 90.5 kg (SD, 14.7), with an average BMI of 30.3 (ranging from 29.0 to 32.7).

**Table 1:** Characteristics of the participants at baseline.

Variables	Total	Control group	50 g/d whole grain group	100 g/d whole grain group
	*N* = 115	*N* = 38	*N* = 38	*N* = 39
Age, mean (SD), y	35.7 (8.9)	35.6 (10.3)	35.3 (7.2)	36.2 (9.1)
Sex, Male, No. (%)	63 (54.8%)	21 (55.3%)	20 (52.6%)	22 (56.4%)
Education level, College, No. (%)	78 (67.8%)	25 (65.8%)	24 (63.2%)	29 (74.4%)
Marital status, Married, No. (%)	85 (73.9%)	27 (71.1%)	28 (73.7%)	30 (76.9%)
Smoking status, yes, No. (%)	42 (36.5%)	16 (42.1%)	15 (39.5%)	11 (28.2%)
Drinking status, yes, No. (%)	72 (62.6%)	25 (65.8%)	23 (60.5%)	24 (61.5%)
Systolic blood pressure, mm Hg	129 (16.1)	129 (17.8)	129 (16.4)	129 (14.3)
Diastolic blood pressure, mm Hg	84.5 (11.2)	86.3 (12.5)	85.3 (11.6)	82.0 (9.14)
Physical activity, METs per week	14.7 (23.5)	20.7 (35.1)	11.5 (14.3)	11.9 (14.5)
Sedentary time, hours per day	6.26 (2.94)	6.07 (3.09)	6.28 (2.86)	6.43 (2.94)
Weight, kg	90.5 (14.7)	90.2 (15.2)	90.4 (14.5)	90.8 (14.8)
Body fat mass, kg, median (IQR)	31.2 [28.3; 36.2]	30.2 [27.6; 34.5]	31.3 [27.8; 36.1]	31.2 [29.4; 38.2]
Waist circumference, cm, median (IQR)	104 [98.8; 112]	102 [97.0; 111]	103 [98.7; 111]	106 [101; 113]
Body lean mass, kg	54.1 (10.7)	54.4 (10.9)	53.8 (10.00)	54.2 (11.4)
Body mass index, kg/m^2^, median (IQR)	30.3 [29.0; 32.7]	29.6 [28.8; 31.9]	30.4 [29.2; 33.4]	30.5 [29.2; 33.0]
Body fat percent, %	36.7 (6.53)	35.5 (6.30)	36.9 (6.87)	37.7 (6.40)
Area of abdominal visceral fat, cm^2^	155 (40.7)	149 (39.3)	155 (44.4)	162 (38.0)
Glucose level, mmol/L, median (IQR)	5.70 [5.34; 6.12]	5.71 [5.40; 6.20]	5.58 [5.21; 5.95]	5.73 [5.42; 6.13]
GSP, umol/L	188 (30.7)	194 (29.0)	184 (39.4)	187 (20.9)
HbA1c%, median (IQR)	4.95 [4.72; 5.24]	4.98 [4.70; 5.24]	4.92 [4.75; 5.35]	4.99 [4.68; 5.22]
HOMA-IR, median (IQR)	5.30 [3.80; 8.05]	5.05 [3.45; 7.00]	5.90 [4.12; 9.52]	5.10 [3.85; 7.65]
Total cholesterol, mmol/L	5.62 (1.20)	5.71 (1.52)	5.49 (1.12)	5.66 (0.93)
Triglycerides, mmol/L, median (IQR)	1.52 [1.00; 2.34]	1.37 [0.90; 2.13]	1.71 [1.14; 2.30]	1.59 [1.08; 2.41]
High-density lipoprotein cholesterol, mmol/L	1.45 (0.26)	1.46 (0.29)	1.41 (0.25)	1.49 (0.23)
Low-density lipoprotein cholesterol, mmol/L	3.59 (0.90)	3.62 (1.05)	3.47 (0.85)	3.69 (0.80)
TyG	8.89 (0.68)	8.88 (0.80)	8.88 (0.70)	8.92 (0.54)
Uric acid, umol/L	459 (109)	479 (95.8)	452 (127)	445 (102)
Homocysteine, umol/L, median (IQR)	13.7 [11.0; 17.5]	15.2 [12.1; 18.8]	13.0 [9.92; 18.0]	13.3 [11.2; 16.4]

Continuous variables are presented as means ± SD or median (IQR). Categorical variables are presented as No. (%).

IQR, interquartile range; GSP, glycated serum protein; HbA1c, glycated hemoglobin; BMI, body mass index; HOMA-IR, Homeostatic Model Assessment for Insulin Resistance; TyG, triglyceride-glucose index.

### 3.2 The effects of whole grain on weight loss

**Figure 2:**
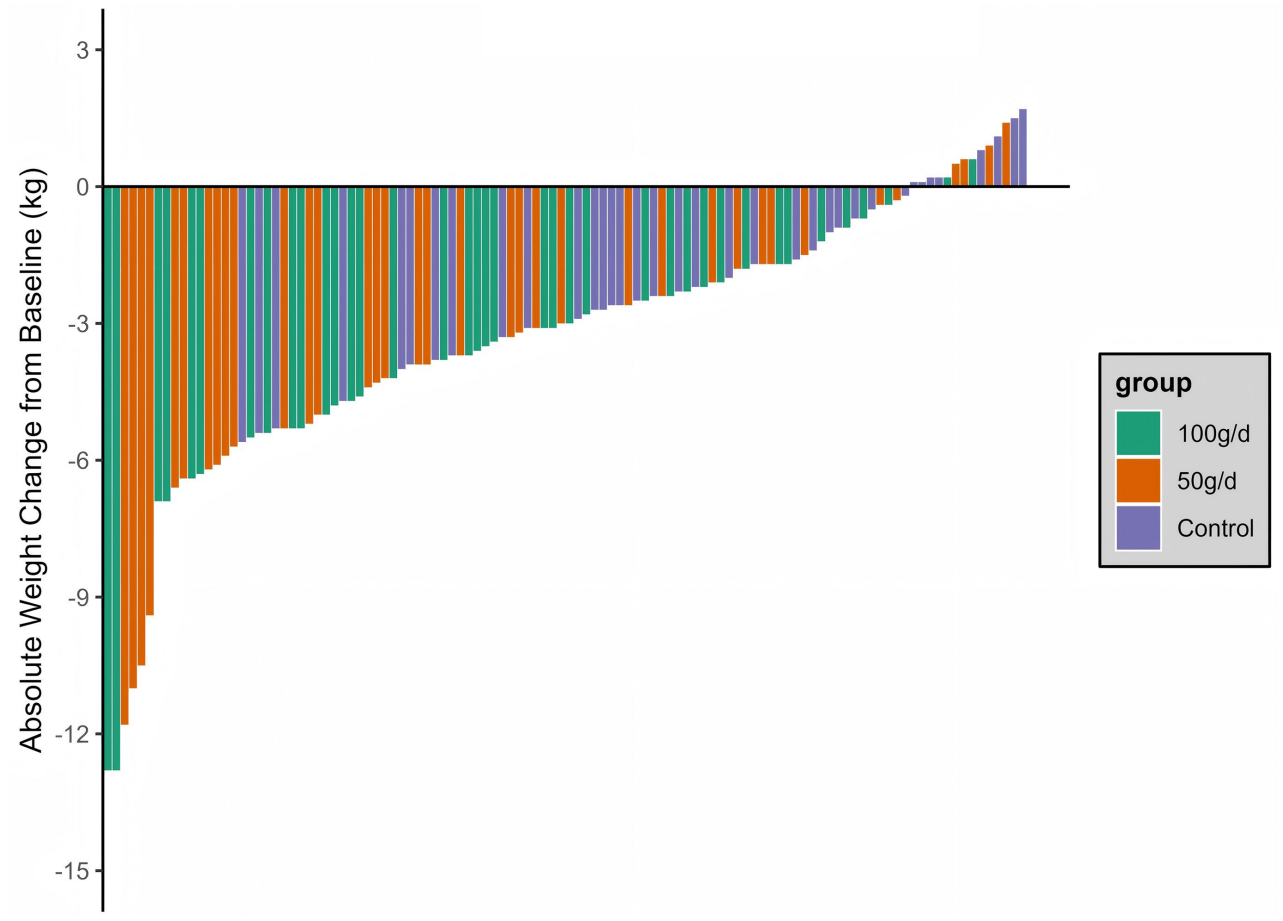
Weight change from baseline to 12 weeks.

As demonstrated in Figure 2, both the 50 g/d and 100 g/d whole grain groups exhibited a statistically significant relative reduction in body weight during the 12-week trial. Following adjustment for confounders, the mean weight change from baseline to 12 weeks between the 50 g/d whole grain group and the control group was −2.0 kg (95% CI, −3.1 to −0.8), and −1.7 kg (95% CI, −2.7 to −0.6) between the 100 g/d whole grain group and the control group (Table 2). The mean weight change between the 100 g/d whole grain group and the 50 g/d whole grain group did not exhibit a statistically significant difference (0.3 kg (95% CI, −0.9 to 1.5)). The BMI also exhibited a decrease from baseline in all three groups, with a more pronounced decline observed in the 50 g/d and 100 g/d whole grain groups. The mean BMI change was −0.7 (95% CI, −1.1 to −0.3) (50 g/d whole grain group vs. control group) and −0.6 (95% CI, −1.0 to −0.2) (100 g/d whole grain group vs. control group) (Table 2). The 100 g/d whole grain group and the 50 g/d whole grain group exhibited comparable reductions in BMI from the baseline, with a mean difference of 0.1 (95% CI, −0.3 to 0.6) (Table 2). Furthermore, a decline in waist circumference was observed in both the 50 g/d and 100 g/d whole grain groups from the baseline. However, only the 50 g/d whole grain group exhibited a statistically significant difference when compared to the control group, with a mean effect size of −1.9 cm (−3.5 to −0.3) (Table 2).

**Table 2:** Effects of whole grain on weight loss and body composition.

Outcome	Control group (*n* = 38)	50 g/d whole grain group (*n* = 38)	100 g/d whole grain group (*n* = 39)	Group-by-time interaction effect	50 g/d whole grain group vs. control group		100 g/d whole grain group vs. control group		100 g/d whole grain group vs. 50 g/d whole grain group	
					Adjusted mean difference (95% CI)	*p* value	Adjusted mean difference (95% CI)	*p* value	Adjusted mean difference (95% CI)	*p* value
Body weight, kg										
Week 6	89.4 (0.3)	88.1 (0.3)	88.4 (0.3)	0.057	−1.2 (−2.2, −0.3)	0.009	−1.0 (−1.9, −0.1)	0.034	0.2 (−0.7, 1.2)	0.606
Week 12	88.1 (0.3)	86.1 (0.5)	86.4 (0.4)		−2.0 (−3.1, −0.8)	0.001	−1.7 (−2.7, −0.6)	0.001	0.3 (−0.9, 1.5)	0.635
Body-mass index, kg/m^2^										
Week 6	31.1 (0.1)	30.7 (0.1)	30.8 (0.1)	0.044	−0.5 (−0.8, −0.1)	0.006	−0.4 (−0.7, 0.0)	0.028	0.1 (−0.2, 0.4)	0.535
Week 12	30.7 (0.1)	29.9 (0.2)	30.1 (0.2)		−0.7 (−1.1, −0.3)	0	−0.6 (−1.0, −0.2)	0.001	0.1 (−0.3, 0.6)	0.591
Waist circumference, cm										
Week 6	105.9 (0.5)	105.0 (0.4)	105.6 (0.4)	0.037	−1.0 (−2.2, 0.3)	0.123	−0.3 (−1.6, 0.9)	0.614	0.6 (−0.4, 1.7)	0.245
Week 12	105.9 (0.5)	103.0 (0.6)	103.9 (0.5)		−1.9 (−3.5, −0.3)	0.018	−1.1 (−2.5, 0.3)	0.14	0.9 (−0.7, 2.4)	0.275
Body fat mass, kg										
Week 6	32.5 (0.4)	31.9 (0.3)	32.1 (0.3)	0.004	−0.6 (−1.6, 0.4)	0.223	−0.4 (−1.5, 0.7)	0.51	0.2 (−0.6, 1.1)	0.587
Week 12	31.7 (0.4)	30.1 (0.4)	30.5 (0.3)		−1.6 (−2.7, −0.5)	0.005	−1.2 (−2.3, −0.2)	0.021	0.4 (−0.8, 1.5)	0.527
Body fat percent, %										
Week 6	36.3 (0.4)	36.0 (0.3)	36.3 (0.4)	0.001	−0.3 (−1.2, 0.7)	0.605	0.0 (−1.1, 1.1)	0.981	0.3 (−0.6, 1.2)	0.553
Week 12	35.9 (0.4)	34.4 (0.5)	34.7 (0.4)		−1.4 (−2.6, −0.2)	0.019	−1.2 (−2.3, −0.1)	0.036	0.2 (−1.0, 1.5)	0.701
Body lean mass, kg										
Week 6	53.5 (0.3)	53.3 (0.2)	53.4 (0.2)	0.539	−0.2 (−1.0, 0.5)	0.532	−0.1 (−0.8, 0.6)	0.754	0.1 (−0.5, 0.7)	0.698
Week 12	53.0 (0.3)	52.9 (0.2)	51.8 (1.0)		−0.1 (−0.8, 0.6)	0.733	−1.2 (−3.2, 0.9)	0.259	−1.1 (−3.3, 1.2)	0.348
Area of abdominal visceral fat, cm^2^										
Week 6	155.2 (2.0)	150.2 (1.6)	147.7 (3.6)	0.054	−5.0 (−10.1, 0.1)	0.056	−7.5 (−15.8, 0.7)	0.073	−2.5 (−9.2, 4.1)	0.458
Week 12	150.8 (2.0)	141.8 (2.4)	140.4 (3.8)		−9.0 (−15.2, −2.7)	0.005	−10.4 (−19.0,-1.8)	0.018	−1.4 (−9.4, 6.6)	0.733

### 3.3 The effects of whole grain on body composition

The present study examined the effects of whole grain consumption on body fat mass in a 12-week trial. The results demonstrated a decrease in body fat mass across all three groups. Following adjustment for confounders, the mean body fat mass change between the 50 g/d whole grain group and the control group was found to be −1.6 kg (95% CI, −2.7 to −0.5) and between the 100 g/d whole grain group and the control group was found to be −1.2 kg (95% CI, −2.3 to −0.2). Furthermore, participants in the 50 g/d and 100 g/d whole grain groups exhibited comparable reductions in body fat percentage and the area of abdominal visceral fat from the baseline. Despite a decline in body lean mass being observed in all three groups, no statistical differences were identified between them (see Table 2).

### 3.4 The effects of whole grain on glucose and lipid metabolism

A comparison was made of glucose and lipid metabolism-related indicators across the three groups, as presented in Table 3. The intervention group demonstrated a reduction in fasting blood glucose levels and glycated serum protein (GSP) during the 6-week and 12-week follow-up periods. A statistically significant decrease of −0.6 mg/dL (95% CI, −0.9 to −0.4) in fasting blood glucose was observed in the 50 g/d whole grain group, while a decrease of −0.6 mg/dL (95% CI, −0.8 to −0.3) was seen in the 100 g/d whole grain group. These changes were compared with the control group from baseline to 12-week follow-up. The 50 g/d whole grain group demonstrated a decline in HbA1c% and HOMA-IR index in comparison to the control group at both 6 and 12 weeks, with HbA1c% exhibiting a change of −0.6 (95% CI, −1.0 to −0.2) and HOMA–IR changing −1.8 (95% CI, −3.2 to −0.3). The 100 g/d whole grain group demonstrated a decrease in HbA1c% and HOMA-IR index at 12 weeks, with HbA1c% reduced by −0.4 (95% CI, −0.7 to −0.1) and HOMA–IR index decreased by −1.6 (95% CI, −2.7 to −0.5).

**Table 3:** Effects of whole grain on cardiovascular risk factors during 12-week trial period.

Outcome	Control group (*n* = 38)	50 g/d whole grain group (*n* = 38)	100 g/d whole grain group (*n* = 39)	Group-by-Time interaction effect	50 g/d whole grain group vs. control group		100 g/d whole grain group vs. control group		100 g/d whole grain group vs. 50 g/d whole grain group	
					Adjusted mean difference (95% CI)	*p* value	Adjusted mean difference (95% CI)	*p* value	Adjusted mean difference (95% CI)	*p* value
Glucose level, mmol/L										
Week 6	6.1 (0.2)	5.3 (0.2)	5.6 (0.1)	0.482	−0.8 (−1.3, −0.3)	0.001	−0.5 (−0.8, −0.1)	0.007	0.3 (−0.1, 0.8)	0.112
Week 12	5.9 (0.1)	5.2 (0.1)	5.3 (0.1)		−0.6 (−0.9, −0.4)	0	−0.6 (−0.8, −0.3)	0	0.1 (−0.1, 0.3)	0.331
GSP, umol/L										
Week 6	200.1 (3.5)	180.1 (3.7)	189.7 (1.8)	0.403	−19.9 (−30.5, −9.3)	0	−10.4 (−18.5, −2.3)	0.012	9.6 (1.9, 17.3)	0.015
Week 12	194.1 (3.3)	179.5 (2.3)	184.1 (1.9)		−14.6 (−23.3, −5.9)	0.001	−10.0 (−17.6, −2.4)	0.01	4.6 (−1.2, 10.4)	0.119
HbA1c%										
Week 6	5.7 (0.1)	5.4 (0.1)	5.6 (0.0)	0.068	−0.3 (−0.6, −0.1)	0.019	−0.1 (−0.3, 0.1)	0.433	0.2 (0.1, 0.4)	0.004
Week 12	5.9 (0.1)	5.3 (0.1)	5.5 (0.1)		−0.6 (−1.0, −0.2)	0.001	−0.4 (−0.7, −0.1)	0.01	0.2 (−0.2, 0.5)	0.272
HOMA–IR index value										
Week 6	6.4 (0.8)	4.4 (0.4)	5.0 (0.3)	0.791	−2.0 (−3.6, −0.3)	0.018	−1.4 (−2.9, 0.2)	0.087	0.6 (−0.3, 1.5)	0.22
Week 12	6.1 (0.5)	4.3 (0.7)	4.5 (0.4)		−1.8 (−3.2, −0.3)	0.017	−1.6 (−2.7, −0.5)	0.005	0.2 (−1.0, 1.4)	0.767
Triglycerides, mmol/L										
Week 6	2.1 (0.3)	1.7 (0.1)	1.6 (0.1)	0.715	−0.5 (−1.0, 0.1)	0.087	−0.5 (−1.1, 0.0)	0.061	−0.1 (−0.3, 0.2)	0.667
Week 12	1.8 (0.1)	1.4 (0.1)	1.4 (0.1)		−0.4 (−0.8, 0.0)	0.039	−0.4 (−0.7, −0.1)	0.023	0.0 (−0.3, 0.3)	0.947
Total cholesterol, mmol/L										
Week 6	5.6 (0.1)	5.1 (0.1)	5.0 (0.1)	0.466	−0.5 (−0.8, −0.2)	0.001	−0.6 (−1.0, −0.2)	0.001	−0.1 (−0.5, 0.3)	0.592
Week 12	5.4 (0.1)	5.0 (0.1)	4.8 (0.1)		−0.4 (−0.7, 0.0)	0.046	−0.6 (−0.9, −0.2)	0.005	−0.2 (−0.6, 0.2)	0.373
High-density lipoprotein cholesterol, mmol/L										
Week 6	1.5 (0.0)	1.4 (0.0)	1.4 (0.0)	0.693	−0.1 (−0.2, 0.0)	0.127	0.0 (−0.1, 0.1)	0.691	0.0 (−0.1, 0.0)	0.12
Week 12	1.4 (0.0)	1.3 (0.0)	1.4 (0.0)		0.0 (−0.1, 0.0)	0.253	0.0 (−0.1, 0.1)	0.791	0.0 (−0.1, 0.0)	0.371
Low-density lipoprotein cholesterol, mmol/L										
Week 6	3.7 (0.1)	3.5 (0.1)	3.7 (0.1)	0.030	−0.5 (−0.5, 0.0)	0.075	0.0 (−0.3, 0.3)	0.935	0.2 (0.0, 0.4)	0.032
Week 12	3.5 (0.1)	3.4 (0.1)	3.4 (0.1)		−0.1 (−0.3, 0.1)	0.465	−0.1 (−0.4, 0.2)	0.459	0.0 (−0.3, 0.2)	0.91
TyG										
Week 6	9.0 (0.1)	8.8 (0.1)	8.7 (0.1)	0.427	−0.2 (−0.4, 0.0)	0.017	−0.3 (−0.5, −0.1)	0	−0.1 (−0.1, 0.3)	0.205
Week 12	8.9 (0.0)	8.7 (0.1)	8.7 (0.1)		−0.3 (−0.4, −0.1)	0	−0.3 (−0.4, −0.1)	0	0.0 (−0.2, 0.2)	0.981
Uric acid, umol/L										
Week 6	453.5 (10.9)	393.6 (7.9)	413.6 (10.2)	0.188	−59.9 (−87.3, −32.5)	0	−39.9 (−70.6, −9.1)	0.011	20.1 (−5.0, 45.2)	0.117
Week 12	427.7 (9.0)	389.9 (8.7)	384.9 (10.9)		−37.8 (−62.8, −12.8)	0.003	−42.8 (−71.2, −14.4)	0.003	−5.0 (−32.4, 22.5)	0.723
Homocysteine, umol/L										
Week 6	16.6 (1.1)	13.4 (0.4)	13.7 (0.5)	0.651	−3.2 (−5.5, −0.8)	0.008	−2.9 (−5.1, −0.7)	0.01	0.3 (−01.5, 1.0)	0.67
Week 12	16.2 (0.6)	14.1 (0.6)	14.6 (0.7)		−2.2 (−4.0, −0.4)	0.017	−1.6 (−3.3, 0.0)	0.048	0.5 (−2.3, 1.2)	0.542
Systolic blood pressure, mm Hg										
Week 6	132.3 (1.7)	129.8 (1.4)	129.1 (1.1)	0.301	−2.5 (−6.7, 1.8)	0.249	−3.2 (−7.2, 0.7)	0.11	−0.7 (−4.2, 2.7)	0.686
Week 12	134.6 (2.2)	128.4 (1.4)	128.7 (1.4)		−6.3 (−11.4, −1.1)	0.018	−6.0 (−11.1, −0.9)	0.02	0.3 (−3.8, 4.3)	0.9
Diastolic blood pressure, mm Hg										
Week 6	85.0 (1.2)	80.1 (2.2)	84.6 (1.1)	0.710	−2.3 (−5.4, 0.9)	0.159	0.4 (−3.0, 3.9)	0.8	4.6 (0.1, 9.0)	0.044
Week 12	85.9 (1.3)	83.2 (1.1)	85.4 (1.2)		−4.6 (−9.0, −0.1)	0.044	0.3 (−3.0, 3.7)	0.848	2.3 (−0.9, 5.4)	0.159
Pulse, beats/min										
Week 6	78.9 (1.2)	77.5 (1.1)	77.6 (1.1)	0.937	−1.4 (−4.7, 1.9)	0.402	−1.3 (−4.6, 1.9)	0.422	0.1 (−3.0, 3.2)	0.963
Week 12	78.0 (0.9)	77.3 (1.2)	77.3 (1.1)		−0.8 (−3.8, 2.3)	0.624	−0.8 (−3.6, 2.1)	0.605	0.0 (−3.1, 3.2)	0.993

Following a 12-week period of intervention, both the 50 g/d and 100 g/d whole grain groups demonstrated a substantial decline in triglycerides and total cholesterol. The mean change for triglycerides was determined to be −0.4 mg/dL (95% CI, −0. 8 to 0.0) in the 50 g/d whole grain group and −0.4 mg/dL (95% CI, −0.7 to −0.1) in the 100 g/d whole grain group, and the mean change in total cholesterol was −0.4 mg/dL (95% CI, −0.7 to 0.0) and −0.6 mg/dL (95% CI, −0.9 to −0.2). A 12-week dietary intervention incorporating whole grains did not demonstrate significant differences in the impact on HDL and LDL levels.

The TyG index was utilised to conduct a comprehensive analysis of the impact of whole grains on blood glucose and lipids. The study results demonstrated that following 6 and 12 weeks of intervention, both intervention groups exhibited a reduction in TyG in comparison to the control group. After 12 weeks of intervention, the mean change value for the 50 g/d whole grain group was −0.3 (95% CI, −0.4 to −0.1), and for the 100 g group was −0.3 (95% CI, −0.4 to −0.1).

Systolic blood pressure was observed to decrease in the 50 g/d and 100 g/d whole grain groups from the baseline during the 12-week trial. When comparing the mean differences between groups, a significant reduction in systolic blood pressure was observed in both the 50 g/d whole grain group (−6.3 mm Hg (95% CI, −11.4 to −1.1)) and 100 g/d whole grain group (−6.0 mm Hg (95% CI, −11.1 to −0.9)). No statistically significant differences were observed in diastolic blood pressure and pulse between the three groups during the 6- and 12-week trial (see Table 3).

### 3.5 The effects of whole grain on blood pressure, serum uric acid and homocysteine levels

As demonstrated in Tables 3, a reduction in both serum uric acid and homocysteine levels was observed in the 50 g/d and 100 g/d whole grain groups from the baseline during the 6- and 12-week trials. After the 12-week trial, a significant reduction in serum uric acid was observed in both the 50 g/d whole grain group (−37.8 umol/L (95% CI, −62.8 to −12.8)) and 100 g/d whole grain group (−42.8 umol/L (95% CI, −71.2 to −14.4)) compared with the control group. Analogous outcomes were ascertained in the serum homocysteine context, exhibiting a mean effect of −2.2 umol/L (95% CI, −4.0 to −0.4) in the 50 g/d whole grain group and −1.6 umol/L (95% CI, −3.3 to −0.0) in the 100 g/d whole grain group.

### 3.6 Dietary intake, physical activity and SF-12 scores during the 12 weeks

Statistically significant reductions in dietary intake were observed in the intervention groups compared with the control group at both 6 and 12 weeks. At week 12, the 50 and 100 g/d whole grain groups decreased by −307 (95% CI, −463 to −151) and −364 kcal/d (95% CI, −538 to −190), respectively, compared to baseline. Furthermore, no statistically significant differences were observed among the three groups regarding physical activity intensity and sedentary time during the 6-week and 12-week follow-up periods. The mental component summary (MCS) scores from the 12-item Short-Form Health Survey Questionnaire (SF-12) were similar among the three groups. However, the physical component summary (PCS) scores showed a certain degree of decline in the 50 g/d and 100 g/d whole grain groups compared to the control group, with only the average change in the 100 g group being statistically significant (−5.6(−9.2, −2.0)) (see Supplementary Table S3).

### 3.7 Adverse events

Apart from mild adverse events, there were no reports of serious or moderate adverse events during the trial period. Mild adverse events, primarily involving the digestive system such as constipation, diarrhea, and dyspepsia, occurred more frequently in the 100 g/d whole grain group compared to the 50 g group; however, the difference was not statistically significant (see Supplementary Table S4).

## 4 Discussion

In a 12-week randomised controlled trial involving 115 adults with obesity, we found that the whole grain intervention (50 and 100 g/d) were associated with a reduction in body weight by a mean change of −2.0 (95% CI, −3.1 to −0.8) and −1.7 kg (95% CI, −2.7 to −0.6), respectively, compared with the control group. The intervention also had a positive effect on several health parameters, including fasting glucose, insulin resistance index, triglycerides, total cholesterol, and blood uric acid and homocysteine levels. However, the effects observed between the 50 g/d and 100 g/d whole grain groups were comparable and not statistically different.

Several previous studies have examined the relationship between whole grain consumption and body weight. Roager et al. conducted a two-arm, 8-week randomised crossover trial comparing whole grain and refined grain diets and found that whole grain consumption was associated with a reduction in body weight compared with refined grain consumption (25). A subsequent meta-analysis of 11 randomised trials (*n* = 919) reported that higher whole grain intake was associated with a greater reduction in weight, with a mean difference of −0.62 kg (95% CI −1.19, −0.05) (16). The present study supports these findings, demonstrating a consistent association between increased whole grain consumption and weight loss. However, a meta-analysis of nine randomised controlled trials (973 participants) found that the summary standardized effect size of the mean difference in weight change between the whole grain intervention group and the control group was −0.049 kg (95% confidence interval −0.297, 0.199, *p* = 0.698), which was not significant (27). It should be noted that the analysis excluded trials with intervention periods of less than 12 weeks, which may have excluded data on the short-term effects of whole grain. In addition, the inclusion of trials where a low-calorie diet was part of the intervention may have confounded the isolated effect of whole grains.

The present study found that, in addition to reducing body weight, whole grain intake was associated with improved blood glucose and lipid metabolism in obese participants. Specifically, whole grain consumption was shown to significantly reduce fasting blood glucose and insulin levels, while improving insulin resistance and reducing triglyceride and total cholesterol levels. These improvements in metabolic markers have been shown to reduce the risk of cardiovascular disease and type 2 diabetes in obese people. Consistent with previous studies (17, 28), our results suggest that whole grain improves blood glucose metabolism. As a high-quality source of carbohydrate, whole grains are rich in fiber, which can slow the absorption of glucose in the intestine and thus reduce the postprandial blood glucose spike. The combined effects of B vitamins, magnesium, and zinc in whole grain on insulin sensitivity together explain the significant association between whole grain intake and improved blood glucose metabolism (29). The current study found that whole grains could reduce triglyceride and total cholesterol levels, which is consistent with the findings of previous research (16, 30). While there is some evidence from studies conducted so far to support the hypothesis (31), the present study did not observe an improvement in high-density lipoprotein and low-density lipoprotein levels as a result of increasing whole grain intake. It is important to note that HDL and LDL levels are influenced by several complex factors, including genetics, diet, and lifestyle habits. Consequently, the interaction of these factors may have obscured the potential effects of the whole grain interventions. The relatively short intervention period of the study is another potential confounding factor, as it was insufficient to detect changes in these lipoproteins within a limited time frame. In recent years, a growing body of research has demonstrated the significant value of TyG indicators in the assessment of insulin resistance (32) and metabolic syndrome (33). The TyG index provides a more accurate reflection of insulin sensitivity by integrating triglyceride and fasting glucose levels. A substantial body of research has demonstrated a strong relationship between the TyG index and cardiovascular disease risk, particularly in individuals with pre-diabetes and obesity (34, 35). In the present study, a significant effect of whole grain intake on the TyG index was observed, suggesting that increasing whole grain intake may improve insulin sensitivity and consequently reduce the risk of cardiovascular disease.

In addition, whole grain was also found to reduce blood uric acid and homocysteine levels in obese people, which is clinically important because hyperuricemia and elevated homocysteine levels are widely recognized as risk factors for cardiovascular and metabolic disease. The observed reduction in serum uric acid following whole grain consumption may be attributed to several mechanisms suggested by previous literature. Firstly, the reduction in serum uric acid observed in our study may be linked to the high content of dietary fiber and antioxidants in whole grains. These components can improve metabolic parameters, and potentially influence uric acid excretion through indirect pathways. In addition, whole grain intake has been shown to reduce inflammatory responses, indirectly influencing uric acid metabolism (36). Minerals such as magnesium and potassium, found in whole grains, have been demonstrated to improve kidney function and further reduce blood uric acid levels (36). Previous studies have shown that whole grain can have a beneficial effect on homocysteine levels in a variety of populations (31, 37). Whole grains are rich in B vitamins, especially folic acid, which are involved in the metabolism of homocysteine in the body and help reduce its accumulation. Whole grains contain antioxidant components, such as polyphenols, which have been shown to reduce oxidative stress and protect vascular endothelial cells. This indirectly reduces homocysteine levels.

The present study set up two intervention dosage groups of 50 g/d and 100 g/d to explore the dose–response relationship of the health effects of whole grains on obese people. Although the intervention effect was shown compared with the control group, no difference was found between the two intervention groups. In 2019, Reynolds et al. systematically reviewed and meta-analyzed the dose–response relationship between whole grains and health outcomes (16). While the dose–response relationship between whole grain intake and key clinical outcomes such as all-cause mortality, coronary heart disease, and type 2 diabetes was delineated based on prospective studies data, the dose–response relationship among whole-grain consumption and body weight could not be established due to the substantial unexplained heterogeneity of clinical trial data (16). Li et al. previously investigated the impact of varying doses of oats (50 and 100 g/d) on weight management in overweight individuals with type 2 diabetes. The study revealed that different doses of oats exhibited comparable weight loss effects during a 30-day intervention period. However, a significant difference was observed, with the 100 g/d oat group demonstrating a substantially greater reduction in weight after a 1-year follow-up (38). According to data on nutrition and monitoring in China (39), the intake of whole grains in the Chinese population was approximately 20.1 g/d, which was significantly below the recommended intake. Given the generally low intake levels of whole grains, an increase of 50 g/d in this study was sufficient to achieve significant health benefits. The intervention duration in this study (e.g., 12 weeks) may have been insufficient to distinguish subtle physiological differences between the various doses. Moreover, the dose gradient established between the 50 g/d and 100 g/d whole grain groups may have been insufficient to statistically trigger observable dose–response differences. Future studies may need to consider longer intervention periods or wider dose intervals.

While the absolute mean weight reduction observed in our study (~1.7 to 2.0 kg) is modest, it represents approximately 2% of baseline body weight, which holds clinical significance. Importantly, evidence from large lifestyle intervention trials and guidelines indicates that even a 3–5% weight loss is sufficient to produce clinically meaningful reductions in triglycerides, blood glucose, and HbA1c, thereby lowering cardiometabolic risk (40, 41). Our findings, achieved through a feasible dietary substitution strategy, approach this threshold and were accompanied by parallel improvements in fasting glucose, TyG index, and lipid profiles. This reinforces that a modest, sustainable weight loss initiated through whole-grain incorporation can yield meaningful health benefits in individuals with obesity.

Our study inevitably has some limitations. Firstly, considering dietary habits and acceptability, the whole grains we intervened with were in the form of mixed whole grains, and we paid more attention to the total amount of intake, which made it impossible to specifically distinguish the effects of each whole grain. Secondly, data on physical activity were collected using questionnaires rather than more accurate methods such as activity trackers. Thirdly, a key limitation is the use of bioelectrical impedance analysis (BIA) for body composition assessment, which provides estimates rather than direct measurements. Specifically in populations with obesity, evidence indicates that BIA may systematically underestimate fat mass and overestimate fat-free mass (42). Nevertheless, the randomised design and identical measurement protocol across all groups support the validity of the between-group comparisons reported in this study. Fourthly, the 12-week intervention period reflected the short-term effects of whole grain on obese people and could not reveal their long-term effects. Therefore, longer intervention studies need to be designed in the future to investigate the long-term effects.

## 5 Conclusion

In adults with obesity, both the 50 g/d and 100 g/d whole grain interventions significantly reduced body weight from baseline to the 12-week follow-up period. In addition, both the whole grain intervention improved glucose and lipid metabolism and reduced blood uric acid and homocysteine levels. No clear dose–response relationship was observed in this study, nor did the degree of improvement in the whole grain intervention group show an increasing trend with higher intervention doses. These findings not only strengthen new evidence that increasing whole grain intake can improve body weight and cardiovascular disease risk factors in obese adults, but also provide a new dietary intervention pathway for weight management in obese populations, and provides a certain scientific basis for clinical practice and public health policy development.

## Data Availability

The raw data supporting the conclusions of this article will be made available by the authors, without undue reservation.
