# Supplementary material for: Effectiveness of whole grain to body weight and cardiometabolic risk in adults with obesity: a parallel randomised controlled trial
Source: Front Nutr. 2026 Mar 11;13:1774209. doi: 10.3389/fnut.2026.1774209 (PMC13013016; doi:10.3389/fnut.2026.1774209)
Supplement: Supplementary file 1 [file Supplementary_file_1.docx]

**Supplementary Tables**

**Supplementary Table S1** Nutrition information of energy and nutrients for the whole grain food (per 100g).

**Supplementary Table S2** Detailed characteristics of the participants at baseline.

**Supplementary Table S3** Changes in dietary intake, physical activity and SF-12 scores during the 12 weeks intervention.

**Supplementary Table S4** Adverse events during intervention during 12-week trial period.

**Supplementary Table S1** Nutrition information of energy and nutrients for the whole grain food (per 100g).

| Energy and nutrients | Quinoa and brown rice | Red rice and brown rice | 50% whole grain buns | 100% whole grain buns |
| --- | --- | --- | --- | --- |
| Energy (kcal) | 153 | 153 | 256.8 | 258.7 |
| Protein (g) | 4.3 | 3.6 | 9.0 | 9.7 |
| Fat (g) | 1.7 | 1.2 | 1.3 | 1.3 |
| Carbohydrates (g) | 30.9 | 33.1 | 51.4 | 51.2 |
| Dietary Fiber (g) | 2.1 | 1.3 | 3.8 | 5.7 |
| Sodium (mg) | 1.7 | 1.7 | 97 | 96 |
| Vitamin B1 (mg) | 0.14 | 0.14 | 0.15 | 0.18 |
| Vitamin B2 (mg) | 0.03 | 0.03 | 0.12 | 0.14 |
| Vitamin B6 (mg) | 0.02 | 0.02 | 0.16 | 0.16 |
| Folic acid μg | 31.3 | 17.4 | 2.5 | 12.1 |
| Calcium (mg) | 21 | 13 | 278 | 283 |
| Potassium (mg) | 104 | 101 | 155 | 236 |
| Magnesium (mg) | 59 | 53 | 553 | 864 |
| Iron (mg) | 1.3 | 1.0 | 31.3 | 41.8 |
| Zinc (mg) | 0.85 | 0.77 | 12.8 | 18.1 |

**Supplementary Table S2** Detailed characteristics of the participants at baseline.

| **Variables** | **Total** | **Control group** | **50 g/d whole grain group** | **100 g/d whole grain group** |
| --- | --- | --- | --- | --- |
|  | **n=115** | **n=38** | **n=38** | **n=39** |
| Age, mean (SD), y | 35.7 (8.9) | 35.6 (10.3) | 35.3 (7.2) | 36.2 (9.1) |
| Sex, No. (%) |  |  |  |  |
| Male | 63 (54.8%) | 21 (55.3%) | 20 (52.6%) | 22 (56.4%) |
| Female | 52 (45.2%) | 17 (44.7%) | 18 (47.4%) | 17 (43.6%) |
| Education level, No. (%) |  |  |  |  |
| High school or below | 14 (12.2%) | 7 (18.4%) | 3 (7.89%) | 4 (10.3%) |
| College | 78 (67.8%) | 25 (65.8%) | 24 (63.2%) | 29 (74.4%) |
| College above | 23 (20.0%) | 6 (15.8%) | 11 (28.9%) | 6 (15.4%) |
| Marital status, No. (%) |  |  |  |  |
| Unmarried | 23 (20.0%) | 10 (26.3%) | 8 (21.1%) | 5 (12.8%) |
| Married | 85 (73.9%) | 27 (71.1%) | 28 (73.7%) | 30 (76.9%) |
| Divorced | 7 (6.09%) | 1 (2.63%) | 2 (5.26%) | 4 (10.3%) |
| Income per month, No. (%) |  |  |  |  |
| < 4000 RMB | 11 (9.57%) | 5 (13.2%) | 2 (5.26%) | 4 (10.3%) |
| 4000 - 8000 RMB | 42 (36.5%) | 18 (47.4%) | 10 (26.3%) | 14 (35.9%) |
| > 8000 RMB | 62 (53.9%) | 15 (39.5%) | 26 (68.4%) | 21 (53.8%) |
| Smoking status, No. (%) |  |  |  |  |
| No | 73 (63.5%) | 22 (57.9%) | 23 (60.5%) | 28 (71.8%) |
| Yes | 42 (36.5%) | 16 (42.1%) | 15 (39.5%) | 11 (28.2%) |
| Drinking status, No. (%) |  |  |  |  |
| No | 43 (37.4%) | 13 (34.2%) | 15 (39.5%) | 15 (38.5%) |
| Yes | 72 (62.6%) | 25 (65.8%) | 23 (60.5%) | 24 (61.5%) |
| Parents' history of hypertension, No. (%) |  |  |  |  |
| No | 64 (55.7%) | 19 (50.0%) | 23 (60.5%) | 22 (56.4%) |
| Yes | 51 (44.3%) | 19 (50.0%) | 15 (39.5%) | 17 (43.6%) |
| Parents' history of diabetic, No. (%) |  |  |  |  |
| No | 88 (76.5%) | 27 (71.1%) | 30 (78.9%) | 31 (79.5%) |
| Yes | 27 (23.5%) | 11 (28.9%) | 8 (21.1%) | 8 (20.5%) |
| Obesity of parents, No. (%) |  |  |  |  |
| No | 69 (60.0%) | 26 (68.4%) | 18 (47.4%) | 25 (64.1%) |
| Yes | 46 (40.0%) | 12 (31.6%) | 20 (52.6%) | 14 (35.9%) |
| Systolic blood pressure, mm Hg | 129 (16.1) | 129 (17.8) | 129 (16.4) | 129 (14.3) |
| Diastolic blood pressure, mm Hg | 84.5 (11.2) | 86.3 (12.5) | 85.3 (11.6) | 82.0 (9.14) |
| Pulse, beats/min | 80.1 (10.7) | 77.8 (10.5) | 81.4 (10.4) | 81.1 (11.1) |
| Physical component summary score | 51.0 [46.0;58.0] | 51.0 [41.0;57.2] | 51.0 [48.0;55.0] | 55.0 [48.0;58.0] |
| Mental component summary score | 52.0 [46.0;55.0] | 55.0 [46.0;59.0] | 49.0 [43.0;55.0] | 52.0 [46.0;55.0] |
| Physical activity, METs per wk | 14.7 (23.5) | 20.7 (35.1) | 11.5 (14.3) | 11.9 (14.5) |
| Sedentary time, hours per day | 6.26 (2.94) | 6.07 (3.09) | 6.28 (2.86) | 6.43 (2.94) |
| Energy intake, kcal/d | 2010[1458-2291] | 2016[1495-2337] | 1982[1434-2264] | 2031[1399-2301] |
| Height, cm | 169 (9.42) | 170 (9.05) | 168 (9.07) | 170 (10.3) |
| Weight, kg | 90.5 (14.7) | 90.2 (15.2) | 90.4 (14.5) | 90.8 (14.8) |
| Body fat mass, kg | 31.2 [28.3;36.2] | 30.2 [27.6;34.5] | 31.3 [27.8;36.1] | 31.2 [29.4;38.2] |
| Body lean mass, kg | 54.1 (10.7) | 54.4 (10.9) | 53.8 (10.00) | 54.2 (11.4) |
| Body mass index, kg/m2 | 30.3 [29.0;32.7] | 29.6 [28.8;31.9] | 30.4 [29.2;33.4] | 30.5 [29.2;33.0] |
| Body fat percent, % | 36.7 (6.53) | 35.5 (6.30) | 36.9 (6.87) | 37.7 (6.40) |
| Area of abdominal visceral fat, cm2 | 155 (40.7) | 149 (39.3) | 155 (44.4) | 162 (38.0) |
| Waist circumference, cm | 104 [98.8;112] | 102 [97.0;111] | 103 [98.7;111] | 106 [101;113] |
| Total cholesterol, mmol/L | 5.62 (1.20) | 5.71 (1.52) | 5.49 (1.12) | 5.66 (0.93) |
| Triglycerides, mmol/L | 1.52 [1.00;2.34] | 1.37 [0.90;2.13] | 1.71 [1.14;2.30] | 1.59 [1.08;2.41] |
| High-density lipoprotein cholesterol, mmol/L | 1.45 (0.26) | 1.46 (0.29) | 1.41 (0.25) | 1.49 (0.23) |
| Low-density lipoprotein cholesterol, mmol/L | 3.59 (0.90) | 3.62 (1.05) | 3.47 (0.85) | 3.69 (0.80) |
| Glucose level, mmol/L | 5.70 [5.34;6.12] | 5.71 [5.40;6.20] | 5.58 [5.21;5.95] | 5.73 [5.42;6.13] |
| Uric acid, umol/L | 459 (109) | 479 (95.8) | 452 (127) | 445 (102) |
| Homocysteine, umol/L | 13.7 [11.0;17.5] | 15.2 [12.1;18.8] | 13.0 [9.92;18.0] | 13.3 [11.2;16.4] |
| GSP, umol/L | 188 (30.7) | 194 (29.0) | 184 (39.4) | 187 (20.9) |
| HbA1c% | 4.95 [4.72;5.24] | 4.98 [4.70;5.24] | 4.92 [4.75;5.35] | 4.99 [4.68;5.22] |
| HOMA-IR | 5.30 [3.80;8.05] | 5.05 [3.45;7.00] | 5.90 [4.12;9.52] | 5.10 [3.85;7.65] |
| TyG | 8.89 (0.68) | 8.88 (0.80) | 8.88 (0.70) | 8.92 (0.54) |

**Supplementary Table S3** Changes in dietary intake, physical activity and SF-12 scores during the 12 weeks intervention.

| **Outcome** | **control group (n=38)** | **50g/d group (n=38)** | **100g/d group (n=39)** | **Group-by-Time interaction Effect** | **50g/d group vs. control group** | | **100g/d group vs. control group** | | **100g/d group vs. 50g/d group** | |
| --- | --- | --- | --- | --- | --- | --- | --- | --- | --- | --- |
|  |  |  |  |  | Adjusted Mean Difference (95% CI) | P value | Adjusted Mean Difference (95% CI) | P value | Adjusted Mean Difference (95% CI) | P value |
| Energy intake, kcal/d | | | | | | | | | | |
| week 6 | 1975(1496-2233) | 1724(1489-1946) | 1674(1316-1984) | 0.659 | -215(-317, -113) | 0.039 | -322(-509, -135) | 0.025 | -126(-261, 9) | 0.056 |
| week 12 | 1941(1458-2291) | 1688(1391-1810) | 1596(1327-1824) |  | -307(-463, -151) | 0.027 | -364(-538, -190) | 0.018 | -73(-169, 23) | 0.061 |
| Carbohydrate, g/d | | | | | | | | | | |
| week 6 | \| 230 (190-265) \| 205 (180-225) \| 195 (160-225) \| 0.415 \| \| --- \| --- \| --- \| --- \| | 205 (180-230) | 195 (160-225) | 0.402 | -22.5 (-46.1, 1.1) | 0.062 | -32.0 (-58.9, -5.1) | 0.020 | -9.5 (-27.3, 8.3) | 0.296 |
| week 12 | 225 (185-260) | 201 (176-221) | 190 (155-220) |  | -18.2 (-41.5, 5.1) | 0.127 | -25.7 (-52.3, 0.9) | 0.058 | -7.5 (-25.1, 10.1) | 0.403 |
| Protein, g/d | | | | | | | | | | |
| week 6 | 84 (69-99) | 88 (73-103) | 86 (73-100) | 0.735 | 3.8 (-6.0, 13.6) | 0.447 | 2.5 (-7.3, 12.3) | 0.618 | -1.3 (-9.2, 6.6) | 0.746 |
| week 12 | 85 (70-100) | 90 (75-105) | 88 (75-102) |  | 4.5 (-5.2, 14.2) | 0.363 | 3.8 (-6.8, 14.4) | 0.483 | -0.7 (-8.5, 7.1) | 0.861 |
| Fat, g/d | | | | | | | | | | |
| week 6 | 74 (62-86) | 66 (56-76) | 63 (53-73) | 0.251 | -7.5 (-16.2, 1.2) | 0.091 | -10.2 (-20.0, -0.4) | 0.041 | -2.7 (-10.0, 4.6) | 0.469 |
| week 12 | 72 (60-84) | 65 (55-75) | 62 (52-72) |  | -6.8 (-15.3, 1.7) | 0.116 | -9.5 (-19.1, 0.1) | 0.053 | -2.7 (-9.8, 4.4) | 0.458 |
| Dietary fibre, g/d | | | | | | | | | | |
| week 6 | 16.0 (12.5-19.5) | 20.0 (17.0-23.5) | 23.0 (20.0-26.5) | 0.002 | 3.8 (1.9, 5.7) | <0.001 | 6.5 (4.3, 8.7) | <0.001 | 2.7 (0.9, 4.5) | 0.003 |
| week 12 | 16.0 (12.5-19.5) | 19.5 (16.5-23.0) | 22.0 (19.0-25.5) |  | 3.3 (1.5, 5.1) | <0.001 | 5.8 (3.8, 7.8) | <0.001 | 2.5 (0.8, 4.2) | 0.004 |
| Physical activity — METs/week | | | | | | | | | | |
| week 6 | 6.6(0-12) | 6.7(0-14) | 7.6(0-16) | 0.784 | 0.2(-3.5, 3.9) | 0.930 | 1.0(-3.4, 5.4) | 0.647 | 0.1(-4.2, 4.4) | 0.978 |
| week 12 | 7.8(0-14) | 6.4(0-10) | 6.8(0-9.5) |  | -0.1(-3.9, 3.6) | 0.938 | 0.2(-4.6, 5.1) | 0.927 | 0.4(-4.0, 4.7) | 0.865 |
| Sedentary time, hours | | | | | | | | | | |
| week 6 | 5.9(0.4) | 7.0(0.4) | 6.0(0.3) | 0.476 | 1.0(-0.1, 2.2) | 0.068 | 0.1(-0.9, 1.0) | 0.891 | -1.0(-2.0, 0.0) | 0.060 |
| week 12 | 6.2(0.4) | 6.8(0.3) | 6.4(0.3) |  | 0.6(-0.5, 1.7) | 0.265 | 0.2(-0.9, 1.3) | 0.710 | -0.4(-1.3, 0.5) | 0.394 |
| SF—12 | | | | | | | | | | |
| PCS | | | | | | | | | | |
| week 6 | 49.0(1.8) | 50.0(1.3) | 49.6(1.6) | 0.041 | 0.6(-4.0, 5.3) | 0.788 | 1.0(-3.3, 5.3) | 0.641 | -0.4(-4.3, 3.6) | 0.849 |
| week 12 | 53.2(1.3) | 47.5(1.3) | 50.0(1.2) |  | -3.2(-6.8, 0.4) | 0.082 | -5.6(-9.2, -2.0) | 0.002 | 2.4(-1.1, 5.9) | 0.171 |
| MCS | | | | | | | | | | |
| week 6 | 49.0(1.6) | 49.1(1.3) | 50.8(1.4) | 0.861 | 0.2(-3.8, 4.1) | 0.935 | 1.8(-2.2, 5.9) | 0.374 | 1.7(-2.2, 5.5) | 0.392 |
| week 12 | 49.4(1.3) | 50.0(1.4) | 50.5(1.6) |  | 0.6(-3.1, 4.3) | 0.736 | 1.1(-2.9, 5.1) | 0.600 | 0.4(-3.9, 4.8) | 0.846 |

PCS, Physical component summary score; MCS, Mental component summary score.

**Supplementary Table S4** Adverse events during intervention during 12-week trial period.

| **Adverse event** | **Patients, No. (%)** | | |
| --- | --- | --- | --- |
|  | **Control group (n=38)** | **50g/d whole grain group (n=38)** | **100g/d whole grain group (n=39)** |
| Serious adverse events | 0 | 0 | 0 |
| Moderate adverse events | 0 | 0 | 0 |
| Mild adverse events | 0 | 2 (5.3) | 7 (17.9) |
| Fatigue | 0 | 0 | 1 (2.6) |
| Dizziness | 0 | 0 | 0 |
| Headache | 0 | 0 | 0 |
| Gastrointestinal adverse events | 0 | 2 | 6 (15.4) |
| Constipation | 0 | 1 | 2 (5.1) |
| Diarrhea | 0 | 0 | 3 (7.7) |
| Dyspepsia | 0 | 1 | 1 (2.6) |
| Any adverse events | 0 | 2 (5.3) | 7 (17.9) |
